# Angiographic collateral status predicts functional outcome and early neurological deterioration in large-vessel occlusion stroke treated with endovascular therapy

**DOI:** 10.3389/fneur.2026.1756513

**Published:** 2026-02-19

**Authors:** Junying Li, Lei Chen, Zhongjiao Lu, Yanhui Li, Yaling Wang, Lanying He, Dan Yang

**Affiliations:** 1 Department of Neurology, West China School of Medicine, Sichuan University, Sichuan University affiliated Chengdu Second People’s Hospital, Chengdu Second People’s Hospital, Chengdu, Sichuan, China; 2 Bai Lian chi Community Health Center, Chengdu, Sichuan, China; 3 Department of Neurology, Renji Hospital, Shanghai Jiao Tong University School of Medicine, Shanghai, China

**Keywords:** acute ischemic stroke, collateral circulation, early neurological deterioration, endovascular treatment, functional outcome, mortality

## Abstract

**Background:**

Despite successful recanalization with endovascular treatment (EVT) for acute ischemic stroke (AIS), many patients experience poor outcomes. While collateral circulation is a known prognostic factor, its dynamic assessment via digital subtraction angiography (DSA) and its relationship to outcomes post-EVT require further investigation.

**Methods:**

This single-center retrospective study analyzed 185 consecutive AIS patients with large vessel occlusion treated with EVT. Collateral status was graded on pre-treatment DSA using the American Society of Interventional and Therapeutic Neuroradiology/Society of Interventional Radiology (ASITN/SIR) scale and categorized as poor, fair, or good. The primary outcome was functional independence (modified Rankin Scale [mRS] score 0–2) at 90 days. Secondary outcomes included early neurological deterioration (END) within 7 days and 90-day mortality.

**Results:**

Patients with good collaterals had significantly higher rates of functional independence (66.1%) compared to those with fair (45.9%) and poor collaterals (13.2%). Conversely, the incidences of END (1.8% vs. 8.2% vs. 23.5%) and 90-day mortality (1.8% vs. 11.5% vs. 27.9%) were progressively higher in the good, fair, and poor collateral groups, respectively. Multivariable logistic regression confirmed that a higher collateral score was an independent predictor of good functional outcome and was independently associated with a lower risk of END and mortality.

**Conclusion:**

DSA-assessed collateral status is a strong independent predictor of 90-day functional outcome, END, and mortality in AIS patients following EVT. Robust pretreatment collaterals are associated with markedly improved recovery and survival, highlighting the critical prognostic value of collateral assessment in guiding treatment and patient management.

## Introduction

Endovascular treatment (EVT) is now established as the standard therapy for acute ischemic stroke (AIS) caused by large-vessel occlusion (LVO) within 24 h (1, 2), with multiple randomized trials demonstrating substantial reductions in disability and mortality (3). However, even after technically successful recanalization, nearly half of patients fail to achieve functional independence at 3 months (4). Notably, considerable outcome variability persists among patients with similar occlusion sites, baseline characteristics, and reperfusion success. This highlights the need for reliable, imaging-based biomarkers to refine treatment selection and improve prognostic precision in EVT population.

Collateral circulation is recognized as one of the most critical determinants of ischemic tissue fate (5, 6). Robust collaterals maintain residual perfusion to the penumbra, slow infarct progression, preserve metabolic viability, and enhance the likelihood of favorable neurological recovery (7, 8). Despite this recognized importance, the extent to which collateral integrity continues to influence outcomes after successful EVT remains incompletely understood. Existing studies have yielded conflicting results, with some demonstrating a strong association between good collaterals and improved outcomes (9, 10), while others reported no significant relationship (11, 12).

Emerging evidence further suggests that collateral status may modulate the time dependence of EVT efficacy. Patients with poor collaterals experience substantial declines in outcome with prolonged onset-to-reperfusion times, whereas those with robust collaterals show relative resistance to ischemic delay (13). Although collateral enhancement has been proposed as a therapeutic target, the clinical impact remains uncertain, partly due to heterogeneous collateral assessment methods and variable patient responses (14). Most prior studies have relied on computed tomography angiography (CTA) for collateral evaluation, where CTA-based grading has shown predictive value for post-EVT outcomes (15–17). However, CTA provides only a static snapshot of vascular filling, whereas digital subtraction angiography (DSA) offers dynamic, higher-resolution assessment of collateral flow. Despite these advantages, the prognostic implications of DSA-based collateral grading remain insufficiently explored. Existing evidence is limited, often restricted to either anterior or posterior circulation strokes, and prior studies have reported mixed findings regarding the strength and consistency of these associations (18, 19).

To address these knowledge gaps, the present study systematically evaluated the relationship between DSA-determined collateral status and short-term functional outcomes in AIS patients with LVO undergoing EVT. In addition, we examined the interaction between collateral status and reperfusion success, along with other clinical and imaging predictors of outcome. This work aims to clarify the prognostic value of angiographic collaterals and provide more precise insights into their role in EVT-treated AIS.

## Materials and methods

### Patients

This single-center retrospective study enrolled consecutive patients with AIS who underwent EVT at the Chengdu Second People’s Hospital between January 2023 and May 2025. The inclusion criteria were as follows: (1) aged ≥ 18 years; (2) diagnosis of AIS due to LVO; (3) receipt of reperfusion therapy, including EVT and/or intravenous thrombolysis (IVT); and (4) availability of high-quality imaging without significant motion artifacts. Patients were excluded if: (1) DSA image was incomplete or unavailable; (2) thrombectomy was unsuccessful or performed as a non-emergency procedure. For patients presenting beyond the conventional treatment window, multimodal magnetic resonance imaging (MRI) or computed tomography (CT) perfusion imaging was used to assess eligibility for reperfusion therapy. In accordance with current guidelines, IVT was administered within 4.5 h of symptom onset or last known well time, and EVT was performed within 24 h (20).

### Data collection

Clinical and demographic data were extracted from the stroke center database, including age, sex, and vascular risk factors such as hypertension, diabetes mellitus, hyperlipidemia, coronary artery disease, atrial fibrillation, transient ischemic attacks (TIA), and prior ischemic stroke. Lifestyle factors, including smoking and alcohol consumption, were also recorded. Clinical assessments included admission blood pressure and initial stroke severity, which was evaluated using the National Institutes of Health Stroke Scale (NIHSS) and the modified Rankin Scale (mRS). Both scales were assessed before reperfusion therapy and were defined as the baseline NIHSS and baseline mRS, respectively. Time metrics included onset-to recanalization time and door-to-puncture time (DPT). The site of vessel occlusion and type of reperfusion were documented for each patient. Stroke etiology was determined according to the modified Trial of ORG 10172 in Acute Stroke Treatment (TOAST) classification.

### Treatments

Treatment strategies, including EVT alone or combined EVT and IVT, were determined by the treating clinicians based on individual clinical and imaging profiles. Patients who presented beyond the standard therapeutic time window were considered for reperfusion therapy if multimodal imaging demonstrated a favorable perfusion-diffusion mismatch, in accordance with the 2018 Chinese Guidelines for the Diagnosis and Treatment of Acute Ischemic Stroke. Informed consent was obtained from all patients or their legal representatives prior to treatment, with comprehensive explanation of potential risks, including hemorrhagic complications and financial implications.

### Measurements

DSA was first performed on the contralateral normal side before thrombectomy to identify the occlusion site and evaluate collateral circulation through the circle of Willis, thereby providing a baseline for endovascular intervention. All examinations were conducted using a biplane angiography system (UNIQ Clarity FD20/20, Philips, The Netherlands) equipped with a power injector (8 mL ioversol, injection rate of 6 mL/s, pressure of 200 psi/kg), following a standardized protocol at Chengdu Second People’s Hospital, China. Two experienced neurointerventionalists, each with 10–20 years of clinical experience, independently reviewed all pre-EVT DSA images while blinded to the patients’ clinical data. Collateral circulation was graded according to the American Society of Interventional and Therapeutic Neuroradiology/Society of Interventional Radiology (ASITN/SIR) scale as follows: grade 0: no visible collaterals to the ischemic site; grade 1: slow collaterals to the periphery of the ischemic area with persistent defects; grade 2: rapid collaterals reaching the ischemic boundary with partial defects; grade 3: slow but complete collateral filling of the ischemic bed by the late venous phase; and grade 4: rapid and complete collateral filling of the ischemic bed via retrograde perfusion (21). Posterior circulation collateral status was assessed using the posterior circulation collateral score (PC-CS), which ranges from 0 to 10. For statistical analysis, PC-CS scores were categorized into equivalent ASITN/SIR grades as follows: 0 as grade 0; 1–3 as grade 1; 4–5 as grade 2; 6–8 as grade 3; and 9–10 as grade 4. This categorization represents an equivalent stratification for analytical purposes rather than a direct one-to-one correspondence between the PC-CS and ASITN/SIR grading systems. For further analysis, collateral status was grouped into three categories: poor (grades 0–1), fair (grade 2), and good (grades 3–4). In cases of disagreement between the two neurointerventionalists, a consensus was reached through joint review. Excellent inter-rater reliability was confirmed on a training dataset prior to independent assessments (intraclass correlation coefficient > 0.90). Functional outcomes at 90 days were assessed using the mRS through face-to-face visits or telephone interviews with patients or family members. An mRS score of 0–2 was defined as a good functional outcome.

### Evaluation of recanalization

The reperfusion after EVT was assessed by an experienced neurointerventionist with over 10 years of experience, using the modified Treatment in Cerebral Infarction scale (mTICI, score 0/1/2a/2b/3), and the mTICI 0/1/2a was defined as unsuccessful reperfusion, the mTICI 2b/3 as successful reperfusion (22), indicating restoration of antegrade perfusion in more than 50% of the affected vascular territory.

### Ethical approval

The study was approved by the Ethics Committee of Chengdu Second People’s Hospital. Written informed consent was obtained from all patients or their legal representatives, confirming their agreement to underdo EVT with or without IVT. All procedures were conducted in accordance with the ethical standards of the institutional research committee and the principles of the Declaration of Helsinki.

### Study outcomes

The primary outcome has the functional status at 90 days after stroke, assessed by the mRS. Patients were categorized into two groups: good outcome (mRS ≤ 2) and poor outcome (mRS > 2) groups. The secondary outcome was the occurrence of early neurological deterioration (END) within 7 days after reperfusion treatment, defined as an increase of ≥4 points in the NIHSS score or a ≥1 point decrease in the level of consciousness subscore within 7 days post-recanalization (23). Safety outcomes included any intracranial hemorrhage (ICH) and all-cause mortality rate within 90 days.

### Statistical analysis

The normality of continuous variables was assessed using the Kolmogorov–Smirnov test. Normally distributed data were expressed as mean ± standard deviation (SD), whereas non-normally distributed data were reported as median [interquartile range (IQR)]. Categorical variables were compared among collateral grade groups using the chi-square test or Fisher’s exact test when appropriate. Continuous variables were compared using one-way ANOVA or the Kruskal-Wallis test, depending on data distribution. When a significant overall difference was observed across collateral grades, pairwise *post hoc* comparisons were conducted using the Wilcoxon rank-sum test. Variables with *p* < 0.05 in univariable analyses were entered into a multivariable logistic regression model (backward stepwise method) to identify independent predictors of favorable outcomes. All statistical tests were two-sided, and *p* < 0.05 was considered statistically significant. Data analyses were performed using SPSS version 23.0 (IBM, Chicago, IL, USA), and graphical outputs were generated using GraphPad Prism version 9.5.1 (GraphPad Software, San Diego, CA, USA).

## Results

### Patient characteristics

**Figure 1:**
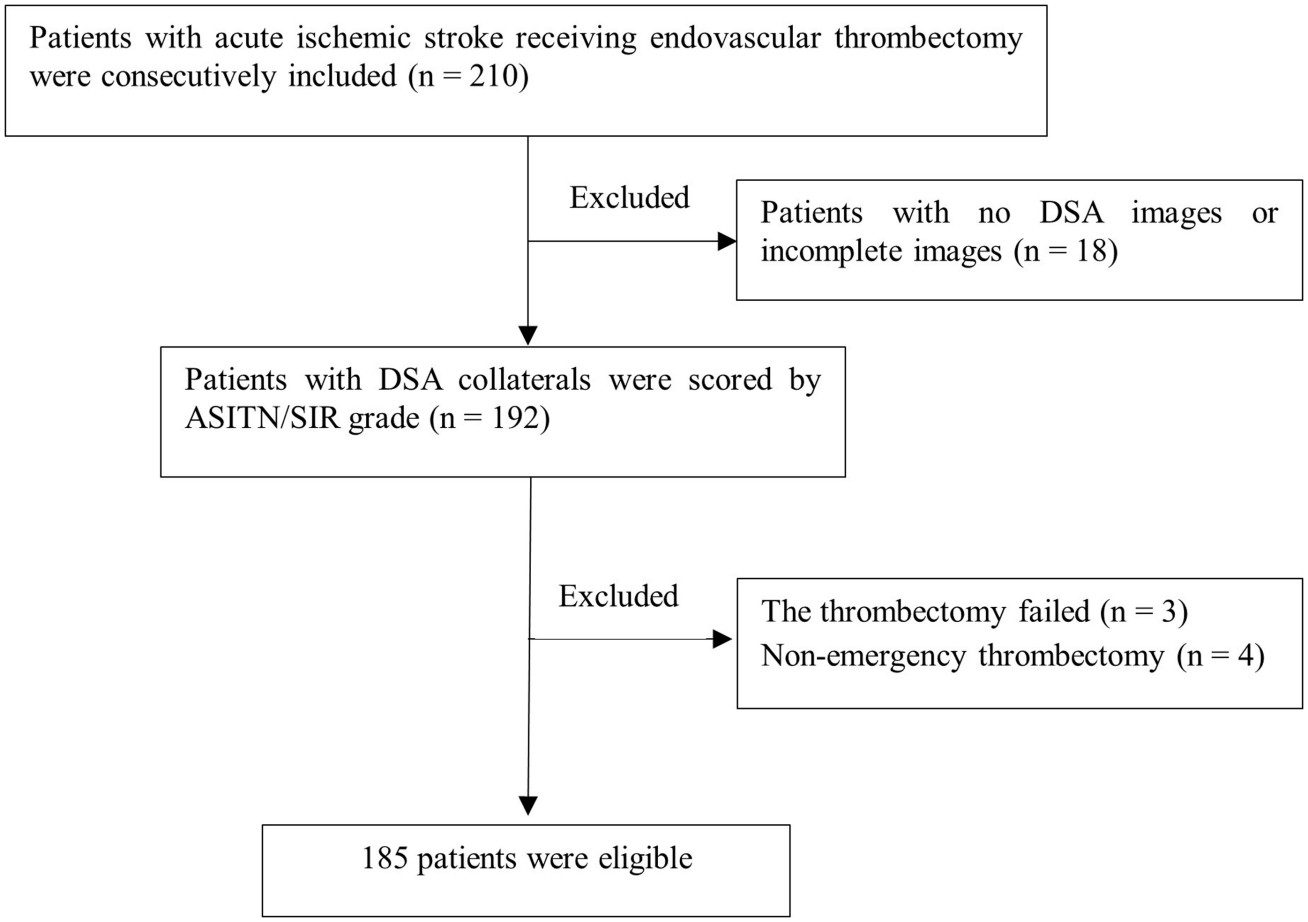
Flowchart of patient selection. DSA, digital subtraction angiography; ASITN/SIR, the American Society of Interventional and Therapeutic Neuroradiology/Society of Interventional Radiology.

A total of 210 patients with AIS who underwent EVT were initially enrolled. After excluding 18 patients with missing or incomplete DSA images, 3 patients with failed thrombectomy, and 4 patients who underwent non-emergency thrombectomy, 185 patients (87 males and 98 females) were included in the final analysis (Figure 1). As shown in Table 1, patients were categorized into three groups based on DSA collateral grades: poor (*n* = 68, 36.7%), fair (*n* = 61, 33.0%), and good (*n* = 56, 30.3%). Significant differences were observed among the three groups for age (78 [69.25, 83.75] vs. 72 [65, 81] vs. 71.5 [65, 80] years, *p* = 0.016), baseline NIHSS (17 [13, 21] vs. 14 [8.5, 16] vs. 9 [5, 15.75], *p* < 0.001), baseline mRS (5 [4, 5] vs. 4 [4, 5] vs. 4 [4, 4.75], *p* < 0.001), DPT (84 [70.25, 135.25] vs. 125 [90.5, 187.0] vs. 145 [112.25, 182.25] minutes, *p* < 0.001), and TOAST classification (*p* = 0.010). However, there were no significant differences among the three groups in terms of sex, vascular risk factors (including smoking, drinking, hypertension, diabetes, hyperlipidemia, coronary artery disease, atrial fibrillation, prior ischemic stroke, and TIA), systolic and diastolic blood pressure, occlusion site, reperfusion type, onset-to-reperfusion time, or rate of successful reperfusion (all *p* > 0.05).

**Table 1:** Comparison of demographic and clinical endpoints among subgroups with good, fair, and poor collaterals.

Variables	Poor collaterals *n* = 68 (36.7%)	Fair collaterals *n* = 61 (33.0%)	Good collaterals *n* = 56 (30.3%)	*p* value
Age, median (Q1, Q3)	78 (69.25, 83.75)	72 (65, 81)	71.5 (65, 80)	0.016*
Sex, male, *n* (%)	28 (41.2)	29 (47.5)	30 (53.6)	0.386
Medical history and risk factors				
Smoke, *n* (%)	12 (17.6)	10 (16.4)	15 (26.8)	0.310
Drinking, *n* (%)	7 (10.3)	9 (14.8)	11 (19.6)	0.340
Hypertension, *n* (%)	38 (55.9)	33 (54.1)	28 (50.0)	0.803
Diabetes, *n* (%)	13 (19.1)	17 (27.9)	13 (23.2)	0.501
Hyperlipidemia, *n* (%)	1 (1.5)	2 (3.3)	3 (5.4)	0.477
Coronary artery disease, *n* (%)	8 (11.8)	6 (9.8)	5 (8.9)	0.866
Atrial fibrillation, *n* (%)	23 (33.8)	13 (21.3)	10 (17.9)	0.090
Previous ischemic stroke, *n* (%)	13 (19.1)	9 (14.8)	6 (10.7)	0.428
Previous TIA, *n* (%)	1 (1.5)	1 (1.6)	2 (3.6)	0.684
Systolic blood pressure, mmHg, mean ± SD	135.72 ± 25.52	140.56 ± 20.86	142.55 ± 22.01	0.232
Diastolic blood pressure, mmHg, mean ± SD	79.12 ± 17.92	79.0 ± 12.74	80.13 ± 12.01	0.902
Baseline NIHSS, median (Q1, Q3)	17 (13, 21)	14 (8.5, 16)	9 (5, 15.75)	<0.001*
Baseline mRS, median (Q1, Q3)	5 (4, 5)	4 (4, 5)	4 (4, 4.75)	<0.001*
Occlusion location, *n* (%)				0.132
Anterior circulation	65 (95.6)	53 (86.9)	48 (85.7)	
Postier circulation	3 (4.4)	8 (13.1)	8 (14.3)	
Reperfusion types, *n* (%)				0.316
EVT	50 (73.5)	41 (67.2)	34 (60.7)	
IVT + EVT	18 (26.5)	20 (32.8)	22 (39.3)	
DPT, median (Q1, Q3)	84.0 (70.25, 135.25)	125.0 (90.5, 187.0)	145.0 (112.25, 182.25)	<0.001*
Onset-to-reperfusion time, median (Q1, Q3)	248.5 (150, 418.75)	280.0 (165.5, 427.0)	299.0 (160.5, 749.5)	0.444
Successful reperfusion (mTICI ≥ 2b), *n* (%)	67 (98.53)	61 (100)	54 (96.43)	0.695
Etiological classification				0.010*
Large artery atherosclerosis, *n* (%)	19 (27.9)	31 (50.8)	35 (62.5)	
Cardio embolism, *n* (%)	46 (67.6)	27 (44.3)	19 (33.9)	
Other known, *n* (%)	2 (2.9)	2 (3.3)	2 (3.6)	
Undetermined, *n* (%)	1 (1.5)	1 (1.6)	0 (0)	
7d END, *n* (%)	16 (23.5)	5 (8.2)	1 (1.8)	0.001*
90d mRS, median (Q1, Q3)	4 (4, 6)	3 (0, 4)	1 (0, 3)	<0.001*
90-day good outcome (mRS score 0–2), *n* (%)	9 (13.2)	28 (45.9)	37 (66.1)	<0.001*
ICH, *n* (%)	13 (19.1)	4 (6.6)	7 (12.5)	0.106
Death, *n* (%)	19 (27.9)	7 (11.5)	1 (1.8)	<0.001*

TIA, transient ischemic attacks; IVT, intravenous thrombolysis; EVT, endovascular thrombectomy; NIHSS, scores on the National Institutes of Health Stroke Scale, mRS, the modified Rankin scale; DPT, door to puncture time; END, early neurological deterioration; ICH, Intracranial hemorrhage; SD, standard deviation; ^*^p < 0.05.

### Efficacy and safety outcomes

**Figure 2:**
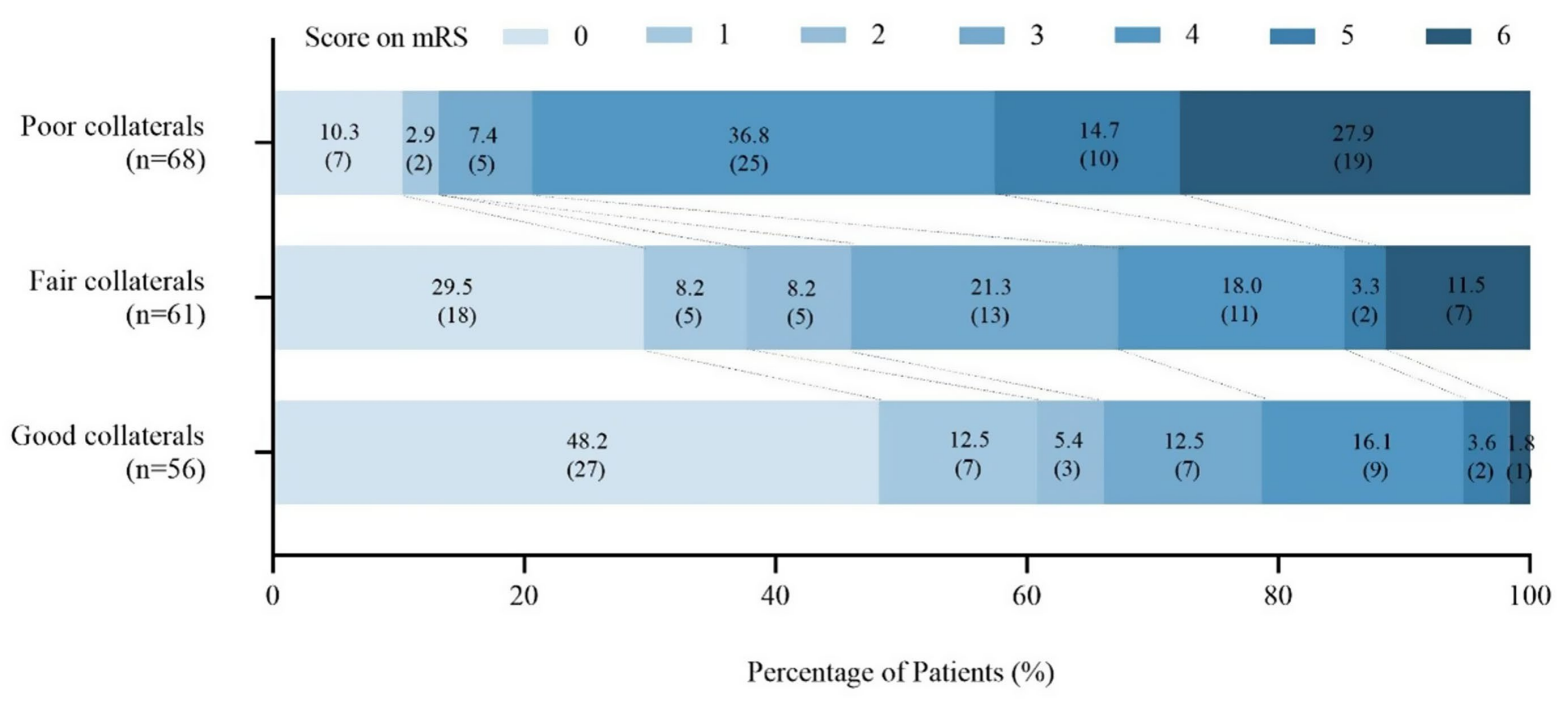
The mRS score at 90 days for patients among three collateral groups. mRS, the modified Rankin scale.

At 90 days after reperfusion therapy, patients with good collaterals had significantly lower median mRS score (1 [0, 3]) compared with those with fair (3 [0, 4]) or poor collaterals (4 [4, 6]) (*p* < 0.001). The proportion of patients achieving functional independence (mRS 0–2) at 90 days also differed significantly among the good, fair, and poor collateral groups (66.1, 45.9, and 13.2%, respectively; *p* < 0.001). Conversely, the incidence of 7-day END decreased progressively from poor to good collaterals (23.5, 8.2, and 1.8%; *p* = 0.001), while the mortality rate at 90 days showed a similar trend (27.9, 11.5, and 1.8%, *p* < 0.001). However, no significant difference was found in the rate of any ICH among the three groups (19.1, 6.6, and 12.5%, *p* = 0.106) (Table 1). The distribution of 90-day mRS scores across the three collateral groups in illustrated in Figure 2.

Among the 185 patients who underwent EVT, 74 patients (40%) achieved a good functional outcome (mRS ≤ 2) at 90 days, while 111 patients (60%) had a poor outcome (mRS > 2). Multivariable logistic regression analysis identified several independent predictors of poor outcome: the presence of diabetes (OR 0.220; 95% CI 0.076–0.635, *p* = 0.005), higher baseline NIHSS score (OR 1.215; 95% CI 1.095–1.348, *p* < 0.001), lower collateral score (OR 0.512; 95% CI 0.324–0.811, *p* = 0.004), occlusion location (OR 0.061; 95% CI 0.012–0.304, *p* = 0.001), and reperfusion type (OR 5.401; 95% CI 2.181–13.374, *p* < 0.001) (Table 2).

**Table 2:** Predictors for poor outcome in patients with EVT.

Variables	Good outcome, mRS 0–2 (*n* = 74, 40%)	Poor outcome, mRS 3–6 (*n* = 111, 60%)	*p* value, univariate	*p* value, multivariate	OR (95% CI)
Age, median (Q1, Q3)	72 (64, 81)	77 (65, 83)	0.068		
Sex, male, *n* (%)	41 (55.4)	46 (41.4)	0.062		
Medical history and risk factors					
Smoke, *n* (%)	18 (24.3)	19 (17.1)	0.230		
Drinking, *n* (%)	13 (17.6)	14 (12.6)	0.350		
Hypertension, *n* (%)	36 (48.6)	63 (56.8)	0.279		
Diabetes, *n* (%)	11 (14.9)	32 (28.8)	0.028*	0.005*	0.220 (0.076–0.635)
Hyperlipidemia, *n* (%)	2 (2.7)	4 (3.6)	0.735		
Coronary artery disease, *n* (%)	6 (8.1)	13 (11.7)	0.429		
Atrial fibrillation, *n* (%)	15 (20.3)	31 (27.9)	0.238		
Previous ischemic stroke, *n* (%)	11 (14.9)	17 (15.3)	0.933		
Previous TIA, *n* (%)	1 (1.4)	3 (2.7)	0.651		
Systolic blood pressure, mmHg, mean ± SD	141.12 ± 20.97	138.23 ± 24.40	0.404		
Diastolic blood pressure, mmHg, mean ± SD	80.46 ± 10.85	78.67 ± 16.65	0.415		
Baseline NIHSS, median (Q1, Q3)	9 (6, 14.25)	16 (13, 20)	<0.001*	<0.001*	1.215 (1.095–1.348)
Baseline mRS, median (Q1, Q3)	4 (4, 5)	5 (4, 5)	<0.001*	0.211	1.785 (0.721–4.423)
Collateral score (ASITN/SIR classification), median (Q1, Q3)	2.5 (2, 3)	1 (1, 2)	<0.001*	0.004*	0.512 (0.324–0.811)
Occlusion location, *n* (%)			0.002*	0.001*	0.061 (0.012–0.304)
Anterior circulation	60 (81.1)	106 (95.5)			
Postier circulation	14 (18.9)	5 (4.5)			
Reperfusion types, *n* (%)			0.001*	<0.001*	5.401 (2.181–13.374)
EVT	40 (54.1)	85 (76.6)			
IVT + EVT	34 (45.9)	26 (23.4)			
DPT, median (Q1, Q3)	144 (104.25, 180.75)	104 (78, 148)	0.001*	0.802	1.000 (0.999–1.002)
Onset-to-reperfusion time, median (Q1, Q3)	236 (133.50, 612.75)	295 (175, 440)	0.441		
Etiological classification			0.073		
Large artery atherosclerosis, *n* (%)	40 (54.1)	45 (40.5)			
Cardio embolism, *n* (%)	30 (40.5)	62 (55.9)			
Other known, *n* (%)	2 (2.7)	4 (3.6)			
Undetermined, *n* (%)	2 (2.7)	0 (0)			

TIA, transient ischemic attacks; IVT, intravenous thrombolysis; EVT, endovascular thrombectomy; NIHSS, scores on the National Institutes of Health Stroke Scale, mRS, the modified Rankin scale; DPT, door to puncture time; ASITN/SIR, the American Society of Interventional and Therapeutic Neuroradiology/Society of Interventional Radiology; SD, standard deviation; OR, odd ratio; 95%CI, 95% confidence interval; ^*^p < 0.05.

Among the 185 patients undergoing EVT, 22 (11.9%) developed END with 7 days, whereas 163 (88.1%) did not. Multivariate logistic regression revealed that collateral score (OR 0.407; 95% CI 0.232–0.714, *p* = 0.002) and onset-to-reperfusion time (OR 1.001; 95% CI 1.000–1.003, *p* = 0.021) were independent predictors of 7-day END (Table 3). At 90 days, 27 patients (14.6%) had died, while 158 (85.4%) were alive. Collateral score (OR 0.443; 95% CI 0.251–0.783, *p* = 0.005) and reperfusion modality (OR 4.122; 95% CI 1.233–13.778, *p* = 0.021) were identified as independent factors of 90-day death (Table 4).

**Table 3:** Predictors for 7- day END in patients with EVT.

Variables	END (*n* = 22, 11.9%)	Non-END (*n* = 163, 88.1%)	*P* value, univariate	*P* value, multivariate	OR (95% CI)
Age, median (Q1, Q3)	78.5 (71.75, 83.5)	73 (65, 82)	0.119		
Sex, male, *n* (%)	10 (45.5)	77 (52.8)	0.875		
Medical history and risk factors					
Smoke, *n* (%)	5 (22.7)	32 (19.6)	0.733		
Drinking, *n* (%)	3 (13.6)	24 (14.7)	1.000		
Hypertension, *n* (%)	13 (59.1)	86 (52.8)	0.576		
Diabetes, *n* (%)	6 (27.3)	37 (22.7)	0.634		
Hyperlipidemia, *n* (%)	0 (0)	6 (3.7)	1.000		
Coronary artery disease, *n* (%)	3 (13.6)	16 (9.8)	0.077		
Atrial fibrillation, *n* (%)	6 (27.3)	40 (24.5)	0.795		
Previous ischemic stroke, *n* (%)	4 (18.2)	24 (14.7)	0.750		
Previous TIA, *n* (%)	0 (0)	4 (2.5)	1.000		
Systolic blood pressure, mmHg, mean ± SD	137.14 ± 25.15	139.69 ± 22.84	0.628		
Diastolic blood pressure, mmHg, mean ± SD	79.18 ± 19.52	79.41 ± 13.88	0.945		
Baseline NIHSS, median (Q1, Q3)	15 (8.75, 20.25)	14 (9, 18)	0.403		
Baseline mRS, median (Q1, Q3)	5 (4, 5)	4 (4, 5)	0.040*	0.104	2.163 (0.852–5.491)
Collateral score (ASITN/SIR classification), median (Q1, Q3)	1 (1, 2)	2 (1, 3)	<0.001*	0.002*	0.407 (0.232–0.714)
Occlusion location, *n* (%)			0.706		
Anterior circulation	21 (95.5)	145 (89.0)			
Postier circulation	1 (4.5)	18 (11.0)			
Reperfusion types, *n* (%)			0.739		
EVT	20 (90.9)	105 (64.4)			
IVT + EVT	2 (9.1)	58 (35.6)			
DPT, min, median (Q1, Q3)	107.5 (75.25, 183.75)	120.0 (83.0, 167.0)	0.540		
Onset-to-reperfusion time, median (Q1, Q3)	357.50 (238.25, 741.25)	269.0 (143.0, 445.0)	0.049*	0.021*	1.001 (1.000–1.003)
Etiological classification			0.940		
Large artery atherosclerosis, *n* (%)	10 (45.5)	75 (46.0)			
Cardio embolism, *n* (%)	11 (50.0)	81 (49.7)			
Other known, *n* (%)	1 (4.5)	5 (3.1)			
Undetermined, *n* (%)	0 (0)	2 (1.2)			

TIA, transient ischemic attacks; IVT, intravenous thrombolysis; EVT, endovascular thrombectomy; NIHSS, scores on the National Institutes of Health Stroke Scale; mRS, the modified Rankin scale; DPT, door to puncture time; ASITN/SIR, the American Society of Interventional and Therapeutic Neuroradiology/Society of Interventional Radiology; SD, standard deviation; OR, odd ratio; 95%CI, 95% confidence interval; ^*^p < 0.05.

**Table 4:** Predictors for 90-day death in patients with EVT.

Variables	Death (*n* = 27, 14.6%)	Alive (*n* = 158, 85.4%)	*p* value, univariate	*p* value, multivariate	OR (95% CI)
Age, median (Q1, Q3)	79 (71, 84)	73 (65, 82)	0.044*	0.225	1.027 (0.984–1.073)
Sex, male, *n* (%)	13 (48.1)	74 (46.8)	0.899		
Medical history and risk factors					
Smoke, *n* (%)	6 (22.2)	31 (19.6)	0.755		
Drinking, *n* (%)	2 (7.4)	25 (15.8)	0.378		
Hypertension, *n* (%)	18 (66.7)	81 (51.3)	0.138		
Diabetes, *n* (%)	8 (29.6)	35 (22.2)	0.395		
Hyperlipidemia, *n* (%)	1 (3.7)	5 (3.2)	1.000		
Coronary artery disease, *n* (%)	2 (7.4)	17 (10.8)	1.000		
Atrial fibrillation, *n* (%)	7 (25.9)	39 (24.7)	0.890		
Previous ischemic stroke, *n* (%)	4 (14.8)	24 (15.2)	0.960		
Previous TIA, *n* (%)	0 (0)	4 (2.5)	1.000		
Systolic blood pressure, mmHg, mean ± SD	134.89 ± 24.76	140.15 ± 22.77	0.275		
Diastolic blood pressure, mmHg, mean ± SD	79.0 ± 18.31	79.45 ± 13.94	0.883		
Baseline NIHSS, median (Q1, Q3)	17 (13, 21)	14 (8, 18)	0.005*	0.971	0.998 (0.896–1.111)
Baseline mRS, median (Q1, Q3)	5 (4, 5)	4 (4, 5)	0.001*	0.056	2.984 (0.973–9.155)
Collateral score (ASITN/SIR classification), median (Q1, Q3)	1 (1, 2)	2 (1, 3)	<0.001*	0.005*	0.443 (0.251–0.783)
Occlusion location, *n* (%)			0.013*	0.328	2.175 (0.458–10.336)
Anterior circulation	24 (88.9)	142 (89.9)			
Postier circulation	3 (11.1)	16 (10.1)			
Reperfusion types, *n* (%)			0.044*	0.021*	4.122 (1.233–13.778)
EVT	23 (85.2)	102 (64.6)			
IVT + EVT	4 (14.8)	56 (35.4)			
DPT, min, median (Q1, Q3)	88 (74, 175)	122.5 (84.5, 168.5)	0.200		
Onset-to-reperfusion time, median (Q1, Q3)	340 (220, 445)	264.5 (148.25, 458.75)	0.322		
Etiological classification			0.654		
Large artery atherosclerosis, *n* (%)	14 (51.9)	71 (44.9)			
Cardio embolism, *n* (%)	13 (48.1)	79 (50.0)			
Other known, *n* (%)	0 (0)	6 (3.8)			
Undetermined, *n* (%)	0 (0)	2 (1.3)			

TIA, transient ischemic attacks; IVT, intravenous thrombolysis; EVT, endovascular thrombectomy; NIHSS, scores on the National Institutes of Health Stroke Scale; mRS, the modified Rankin scale; DPT, door to puncture time; ASITN/SIR, the American Society of Interventional and Therapeutic Neuroradiology/Society of Interventional Radiology; SD, standard deviation; OR, odd ratio; 95%CI, 95% confidence interval; ^*^p < 0.05.

## Discussion

This study retrospectively analyzed 185 AIS patients with LVO who underwent EVT within 24 h of symptom onset, and demonstrated that collateral circulation plays a critical role in shaping both early neurological status and short-term outcomes. Poor collateral flow was strongly associated with unfavorable functional outcomes, higher rates of 7-day END, and increased 90-day mortality. Beyond collateral status, poor functional outcomes were independently associated with diabetes, occlusion site, and type of reperfusion therapy.

A key finding of our study is the robust association between a higher ASITN/SIR collateral score and improved 90-day outcomes. Patients with good outcomes had a markedly higher prevalence of good collaterals compared with those with poor-outcomes group, reinforcing the essential role of collateral perfusion in preserving the ischemic penumbra. These results are consistent with previous and meta-analyses, which show that robust collaterals limit infarct core expansion (11, 24, 25), promote recanalization, reduce distal embolization, and improve neurological recovery (26). Mechanistically, collateral channels maintain perfusion to viable ischemic tissue (5), slowing infarct progression, support delivery of fibrinolytic agents (27, 28), and effectively extend the therapeutic window for reperfusion (5). However, existing literature has not been entirely consistent regarding the prognostic significance of collaterals, partly due to selective inclusion criteria and narrow time windows. In contrast, our study incorporated both anterior and posterior circulation strokes across diverse onset-to-reperfusion intervals, thereby enhancing generalizability. Importantly, only 13.2% of patients with poor collaterals achieved good functional outcomes even after successful revascularization. Given the risks and substantial costs associated with EVT, identifying this subgroup-who may derive minimal benefit and may be predisposed to malignant edema or hemorrhagic transformation-is clinically meaningful (29).

We also observed that patients with lower collateral scores were more likely to experience END and higher rate of 90-day mortality, echoing prior CTA-based reports (30), and reinforcing the concept that poor collateral accelerate infarct-core expansion (12). In addition, onset-to-reperfusion time-an important determinant of collateral integrity-was an independent predictor of END risk in our cohort. Notably, we further demonstrated for that shorter onset-to-reperfusion time significantly reduced END incidence after EVT, supporting the findings from prior studies (31). Although admission-to-puncture time was previously shown to correlate with successful reperfusion (32), this association appeared attenuated in our data. This discrepancy may be partly explained by the influence of retrieval attempts, a major contributor to procedure duration, which may attenuate the effect of admission-to-puncture time on END risk in multivariable models (33). In addition, the time of symptom onset is often uncertain or imprecisely recorded, potentially affecting the observed associations. These findings highlight the complex interplay among procedural timing (33), collateral quality (34), and clinical outcomes (35), and suggest that collateral status may modify the influence of time on EVT efficacy.

Our study found that poor functional outcomes were independently associated with diabetes, occlusion site, and type of reperfusion therapy. Consistent with prior literature, diabetes was significantly related to unfavorable functional recovery. Hyperglycemia contributes to vascular injury (36), disruption of the blood–brain barrier, and increased susceptibility to hemorrhagic transformation, thereby exacerbating ischemic damage and reducing the therapeutic benefits of reperfusion (37, 38). In our cohort, the proportion of posterior circulation occlusion was markedly higher in the good-outcome group (18.9%) than in the poor-outcome group (4.5%). This suggests that, among AIS patients undergoing EVT, those with posterior circulation occlusion may have better 90-day functional outcomes than those with anterior circulation occlusion. These findings align with previous studies demonstrating that clinical outcomes after EVT vary significantly according to occlusion site (39). Unlike most prior investigations that examined either the anterior or posterior circulation in isolation, our study analyzed both regions concurrently, thereby reducing the limitations inherent in evaluating prognosis based on a single vascular territory. Furthermore, our results indicated that functional outcomes differed according to the type of reperfusion therapy (EVT alone vs. EVT + IVT). The proportion of patients receiving combined EVT + IVT was lower in the poor-outcome group (23.4%) compared with the good-outcome group (45.9%). Based on these observations, we hypothesize that IVT prior to EVT may provide additional benefit for 90-day functional prognosis. Collateral circulation can facilitate clearance of microemboli, enhance delivery of endogenous or exogenous fibrinolytic agents to the occlusive site, and improve EVT recanalization rates (28). Accordingly, our findings further support that combined IVT and EVT yields better clinical outcomes than direct EVT alone, and identify the type of reperfusion therapy as an independent predictor of poor functional outcomes in AIS patients undergoing EVT.

Several limitations warrant consideration. First, as a single-center retrospective study spanning a long observational period, selection bias is unavoidable. Only EVT-eligible patients were included, potentially excluding those with severe early ischemic changes or delayed presentation. Larger multicenter prospective studies are needed to validate and extent our findings. Second, variability in angiographic assessment and differences in contrast timing may have influenced collateral grading. Third, angiography is invasive and may not be appropriate for all AIS patients; although CTA is more widely available, it provides structural rather than hemodynamic information and may overestimate collateral grade. Finally, our sample size limited the power of subgroup analyses examining the interaction between onset-to reperfusion time and collateral perfusion.

## Conclusion

In conclusion, poor collateral circulation is strongly associated with unfavorable 90-day functional outcomes, higher rates of 7-day END, and increased 90-day mortality. We also demonstrated that poor functional outcomes were independently related to diabetes, occlusion site, and type of reperfusion therapy received. These findings underscore the prognostic value of collateral assessment and highlight its potential role in guiding treatment selection and risk stratification in AIS. Further multicenter prospective studies are warranted to validate these results and support the integration of collateral evaluation into clinical decision-making.

## Data Availability

The datasets used and/or analyzed during the current study are available from the corresponding author on reasonable request.
